# Correction: Highly active spherical amorphous MoS_2_: facile synthesis and application in photocatalytic degradation of rose bengal dye and hydrogenation of nitroarenes

**DOI:** 10.1039/d3ra90043f

**Published:** 2023-05-31

**Authors:** Namrata Saha, Arpita Sarkar, Abhisek Brata Ghosh, Amit Kumar Dutta, Gopala Ram Bhadu, Parimal Paul, Bibhutosh Adhikary

**Affiliations:** a Department of Chemistry, Indian Institute of Engineering Science and Technology Shibpur Howrah 711 103 India bibhutoshadhikary@yahoo.in +91-33-2668-2916 +91-33-2668-4561-64 ext. 512; b Department of Analytical Science, Central Salt and Marine Chemicals Research Institute Gijubhai, Badheka Marg Bhavnagar 364002 Gujarat India

## Abstract

Correction for ‘Highly active spherical amorphous MoS_2_: facile synthesis and application in photocatalytic degradation of rose bengal dye and hydrogenation of nitroarenes’ by Namrata Saha *et al.*, *RSC Adv.*, 2015, **5**, 88848–88856, https://doi.org/10.1039/C5RA19442C.

In the original manuscript, the authors regret an error in the calculation of the band gap values. The band gap values were not read at *y* = 0, which is a requirement when using the Tauc method.

As such, the authors have provided a new dataset and an updated Fig. 3. Section 3.2 in the original manuscript has been updated accordingly and is included below, and an updated Fig. 3 has been provided.

**Fig. 3:**
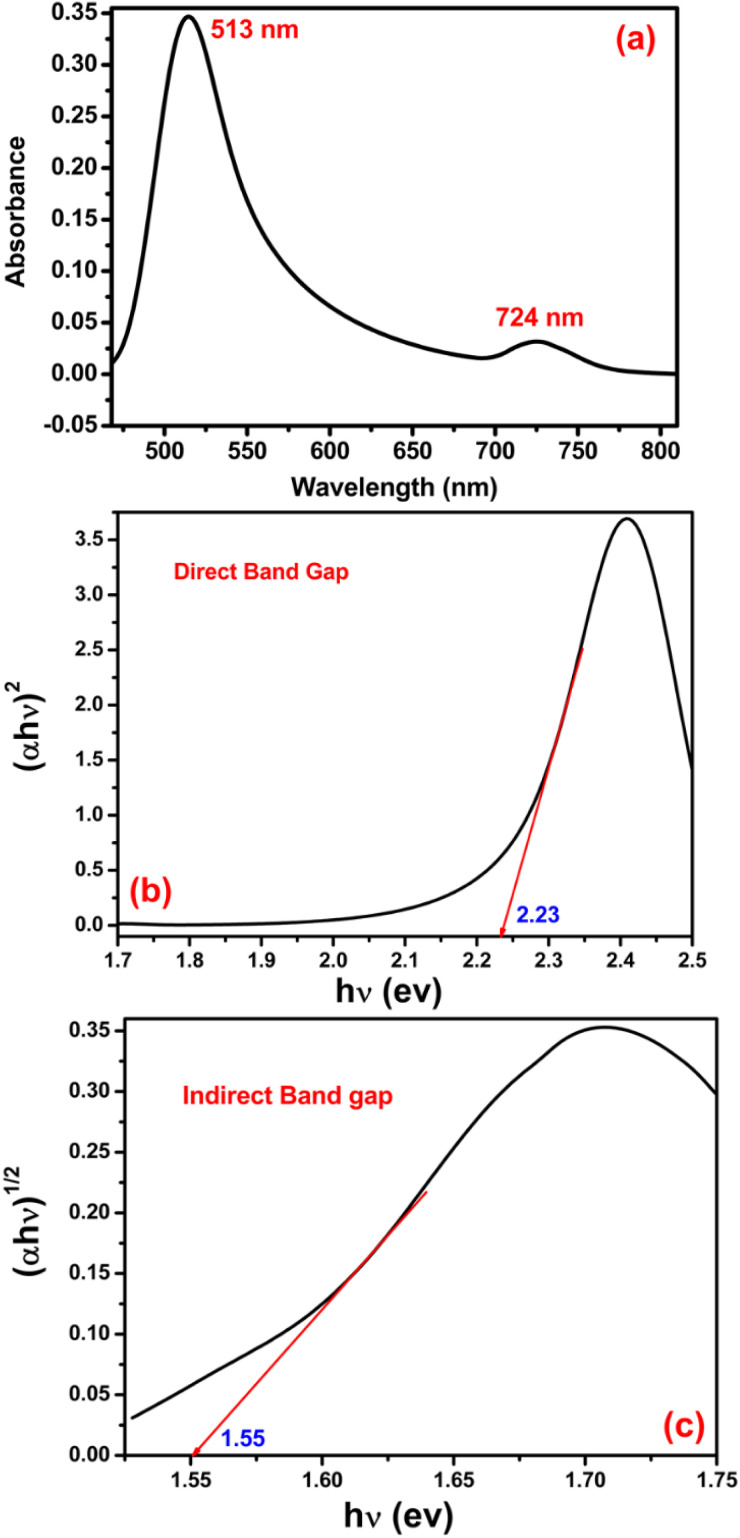
(a) UV-vis absorption spectrum of amorphous MoS_2_. Tauc_Mott plot for (b) direct and (c) indirect bandgap of nanoparticles. The intercept of a solid line with the horizontal axis defines the value of the bandgap.

**3.2. Optical properties**

The typical optical property of amorphous MoS_2_ was investigated by the UV-vis spectroscopic technique and is shown in Fig. 3(a). The room temperature absorption spectrum was recorded by dispersing the sample in toluene. In solution, the first prominent absorption peak is at 513 nm wavelength, which is attributed to transitions involving the direct band gap, and the second absorption shoulder was seen at 724 nm, which corresponds to the indirect band gap of the MoS_2_ amorphous sphere. The presence of both direct and indirect bandgaps simultaneously in the same material was also reported^1^ earlier. Generally, the Tauc plot is used to determine the bandgap of semiconductor materials using the Tauc relation [(*αhν*)^1/*n*^ = *A*(*hν* − *E*_g_)], where, *hν* is the incident photon energy, ‘*A*’ is a constant and ‘*n*’ is the exponent, the value of which is determined by the type of electronic transition causing the absorption and can take the value ½ or 2 depending upon whether the transition is direct or indirect. The direct bandgap of MoS_2_ amorphous sphere is 2.23 eV (Fig. 3(b)), suggesting that MoS_2_ amorphous sphere is a direct bandgap material. Moreover, Fig. 3(c) shows that MoS_2_ amorphous sphere also contains a significant amount of indirect bandgap (1.55 eV) structure, as the TM plot for the indirect bandgap also presents linear sections in that photon energy range.

The presence of both direct and indirect bandgaps simultaneously in the same particles with a smaller indirect bandgap than direct bandgap causes the better use as an efficient catalyst for catalytic reactions under solar light irradiation, provided enough phonons (lattice vibrations) are available to assist the indirect electron transition from the valence band (VB) to the conduction band (CB).

The N_2_ adsorption–desorption isotherms analysis (Fig. S2†) provides further detailed information about the specific surface area of the catalyst. The specific surface area of this sample was calculated to be 32.63 m^2^ g^−1^ by the BET equation. The large surface area of MoS_2_ amorphous sphere is expected to accelerate the photocatalytic reaction by providing more active sites and promoting the separation efficiency of photocarriers.

The scientific conclusions remain unaffected by these changes.

The Royal Society of Chemistry apologises for these errors and any consequent inconvenience to authors and readers.

## Supplementary Material
